# On the Numerical Evaluation of Wall Shear Stress Using the Finite Element Method

**DOI:** 10.1002/cnm.70086

**Published:** 2025-09-12

**Authors:** Jana Brunátová, Jørgen S. Dokken, Kristian Valen‐Sendstad, Jaroslav Hron

**Affiliations:** ^1^ Mathematical Institute Charles University Prague Czechia; ^2^ Bernoulli Institute University of Groningen Groningen the Netherlands; ^3^ Numerical Analysis and Scientific Computing Simula Research Laboratory Oslo Norway; ^4^ Computational Physiology Simula Research Laboratory Oslo Norway

**Keywords:** boundary‐flux evaluation, finite element method, Navier–Stokes equations, stokes flow, wall shear stress

## Abstract

Wall shear stress (WSS) is a crucial hemodynamic quantity extensively studied in cardiovascular research, yet its numerical computation is not straightforward. This work compares WSS results obtained from two different finite element discretizations, quantifies the differences between continuous and discontinuous stresses, and introduces a modified variationally consistent method for WSS evaluation through the formulation of a boundary‐flux problem. Two benchmark problems are considered: a 2D Stokes flow on a unit square and a 3D Poiseuille flow through a cylindrical pipe. These are followed by investigations of steady‐state Navier–Stokes flow in two image‐based, patient‐specific aneurysms. The study focuses on P1/P1 stabilized and Taylor–Hood P2/P1 mixed finite elements for velocity and pressure. WSS is computed using either the proposed boundary‐flux method or as a projection of tangential traction onto first order Lagrange (P1), discontinuous Galerkin first order (DG‐1), or discontinuous Galerkin zero order (DG‐0) space. For the P1/P1 stabilized element, the boundary‐flux and P1 projection methods yielded equivalent results. With the P2/P1 element, the boundary‐flux evaluation demonstrated faster convergence in the Poiseuille flow example but showed increased sensitivity to pressure field inaccuracies in image‐based geometries compared to the projection method. Furthermore, a paradoxical degradation in WSS accuracy was observed when combining the P2/P1 element with fine boundary‐layer meshes on a cylindrical geometry, an effect attributed to inherent geometric approximation errors. In aneurysm geometries, the P2/P1 element exhibited superior robustness to mesh size when evaluating average WSS and low shear area (LSA), outperforming the P1/P1 stabilized element. Projecting discontinuous finite element functions into continuous spaces can introduce artifacts, such as the Gibbs phenomenon. Consequently, it is crucial to carefully select the finite element space for boundary stress calculations, not only in applications involving WSS computations for aneurysms.

## Graphical abstract

We compare a modified variationally consistent boundary‐flux method for wall shear stress evaluation with standard projection technique in aneurysm models and two benchmark examples, demonstrating that the interplay between finite element choice, meshing strategy, and evaluation method has a significant and sometimes counter‐intuitive impact on hemodynamic results.

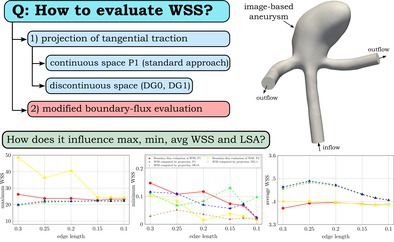

## 1 Introduction

Blood flow‐induced wall shear stress (WSS), the tangential component of stress acting on the endothelium, is essential for maintaining endothelial cell function under normal physiological conditions [1]. Abnormal WSS patterns have been associated with the initiation, progression, and outcomes of vascular diseases such as atherosclerosis [2] and aneurysms [3]. Over the past two decades, medical image‐based computational fluid dynamics (CFD) has played an important role in estimating these stresses [4]. However, there are conflicting results about whether physiologically too high [5] or too low [6] WSS is associated with aneurysm rupture status in large retrospective studies. A hypothesis has been proposed to unify these apparently conflicting results [7], but one of the fundamental problems is that the WSS computations and their mathematical definitions vary considerably [8].

Although many studies have investigated WSS in aneurysms (see recent review articles [9, 10]), a consensus on the optimal numerical approach for its evaluation has yet to be established. A recent study emphasized the lack of standardization in patient‐specific CFD modeling by assigning 26 teams with varying levels of expertise to simulate blood flow using the same set of CT images [11]. The resulting hemodynamic predictions varied widely: interquartile ranges of sac‐averaged WSS reached 56%, only decreasing to below 30% after normalization by parent artery WSS. Sac‐maximum WSS and low shear area showed even greater variability, sometimes differing by an order of magnitude. Importantly, the experience level of the teams did not significantly reduce this variation. Such inconsistencies undermine the trustworthiness required for eventual clinical translation. Therefore, the development of a reliable and reproducible image‐based CFD pipeline is essential. Advancements in this area would enhance the reproducibility of computational studies, facilitate comparisons between studies, and aid in translating computational findings into clinical practice.

The present work focuses specifically on the post‐processing phase of the CFD pipeline, investigating how differences in WSS evaluated via the finite element method (FEM) arise even when the geometry, boundary conditions, viscosity, and so forth, are held fixed. WSS is the tangential force per unit area exerted on the vessel wall. It is intrinsically a scalar quantity; however, it is usually assigned the direction of the force acting on the vessel wall, which has been criticized by Saqr [12]. Various WSS metrics used in the literature have been reported in [8], including its averaged, maximum, and minimum values. In addition to the standard method for WSS computation, we propose and investigate an alternative approach based on the formulation of a boundary flux problem similar to [13, 14, 15]. Furthermore, this study investigates the impact of the choice of finite element spaces on the accuracy of WSS assessment, which has not been shown in previous studies.

All numerical methods are validated against two benchmark problems with known analytical solutions. Subsequently, the same approach is applied to two image‐based patient‐specific aneurysm geometries to demonstrate its ability to handle anatomically relevant situations under simplified flow conditions. Numerical simulations of both Stokes and Navier–Stokes flows employ a stable mixed finite element space: either the P1/P1 element with appropriate stabilization or the Taylor–Hood P2/P1 element. Our open‐source code based on the FEniCS library [16] is used for numerical simulations.

The findings of this study aim to elucidate the numerical aspects of WSS assessment, providing a comparative evaluation of the methods employed and discussing the applicability of each approach. By examining the differences among these techniques, we aim to offer insights into their suitability for accurate and reliable WSS evaluation in image‐based CFD modeling.

## 2 Theory

In this study, we consider incompressible fluid flow, modeled by either the Stokes equations or the Navier–Stokes equations. The FEM will be used for spatial discretization, and a finite difference scheme for temporal discretization. WSS will be assessed in two ways; a projection of the tangential traction force into an appropriate finite element space, and a boundary‐flux evaluation method. Detailed descriptions of both assessment techniques are provided in this section. In the following, we will denote the domain occupied by the fluid as $\Omega \subset {\mathbb{R}}^{n}$, where n=2,3.

### 2.1 Stationary Stokes Flow

The stationary Stokes flow of an incompressible fluid is governed by the following equations

(1a) $$\mathrm{div}\;T\left(v,p\right)+f=0\;\text{in}\;\Omega$$

(1b) $$\;T\left(v,p\right)=-pI+\nu \nabla v\;\text{in}\;\Omega$$

(1c) divv=0inΩ

where v is the velocity and ν kinematic viscosity of the fluid; p denotes the pressure; $T\left(v,p\right)=-pI+\nu \nabla v$ is the Cauchy stress tensor, where I is the identity matrix in dimension 2 or 3; and f denotes any external volume forces. Boundary conditions (BCs) are usually imposed by prescribing the velocity on the boundary (Dirichlet BC), by prescribing traction force acting on the boundary (Neumann BC), or by a combination of the two

(2) $$\mathrm{BCs}\left\{\begin{array}{cc} v=v_{\mathrm{bc}}, & \mathrm{on}\;\Gamma_{D}\\ T\left(v,p\right)n=g & \mathrm{on}\;\Gamma_{N} \end{array}\right.$$

where $\bar{\Gamma_{D}\cup \Gamma_{N}}=\partial \Omega$, $\Gamma_{D}\cap \Gamma_{N}=\emptyset$. When imposing a Dirichlet BC for velocity, it can be applied either in a strong sense [17], which is the common practice, or in a weak sense using Nitsche's method [18, 19, 20]. The choice of whether to impose the BC strongly or weakly will depend on the selection of finite element for velocity and pressure. Additionally, some stabilization terms may be included, depending on the stability requirements of the finite element formulation. The corresponding weak formulation of the problem 1, which incorporates the Nitsche enforcement of boundary conditions, is as follows:

(3) $$\begin{array}{c} \begin{array}{l} \text{Find}\;\left(v,p\right)\in V\times Q\;\text{such that}:\\ \;\int_{\Omega }T:\nabla w\;\mathrm{dx}-\int_{\Gamma_{N}}g\cdot w\;\mathrm{dS}+\int_{\Omega }q\mathrm{div}\;v\;\mathrm{dx}+N_{\Gamma_{D}}\left[\left(v_{\mathrm{bc}};v,p\right),\left(w,q\right)\right]+\\ \;+S\left[\left(v,p\right),\left(w,q\right)\right]=\int_{\Omega }f\cdot w\;\mathrm{dx}\;\forall \left(w,q\right)\in V^{0}\times Q \end{array} \end{array}$$

where we used integration by parts and where $N_{\Gamma_{D}}$ denotes additional terms if BCs on $\Gamma_{D}$ are imposed by Nitsche's method, while S denotes stabilization terms, which will be described in Section 3. In the case of Dirichlet BCs only, the function space for pressure is defined as $Q\overset{\mathrm{def}\;}{=}L_{0}^{2}\left(\Omega \right)$, representing $L^{2}$ functions with zero integral over the domain Ω. When a Neumann BC is included, which will be the case for 3D problems, the function space becomes $Q\overset{\mathrm{def}\;}{=}L^{2}\left(\Omega \right)$. The function space for velocity will be denoted differently when using Nitsche enforcement of BC, $V\overset{\mathrm{def}\;}{=}W^{1,2}\left(\Omega \right)$, and strong enforcement of BC, $V^{v_{\mathrm{bc}}}\overset{\mathrm{def}\;}{=}\left\{v\in W^{1,2}\left(\Omega \right)\;|\;v=v_{\mathrm{bc}}\;\mathrm{on}\;\Gamma_{D}\right\}$.

We will utilize the non‐symmetric Nitsche's method to enforce the Dirichlet BCs [20]. In principle, the non‐symmetric Nitsche's method does not require a penalization term like its symmetric variant; however, it has been shown that for Navier slip BC, using the penalization term can significantly improve the accuracy of the quantities of interest [21]. The non‐symmetric Nitsche method with penalization parameter for enforcing Dirichlet BCs on boundary $\Gamma_{D}$ follows

(4) $$\begin{array}{c} N_{\Gamma_{D}}\left[\left(v_{\mathrm{bc}};v,p\right),\left(w,q\right)\right]=-\int_{\Gamma_{D}}\left(T\left(v,p\right)n\right)\cdot w\;\mathrm{dS}+\int_{\Gamma_{D}}\left(T\left(w,q\right)n\right)\cdot \left(v-v_{\mathrm{bc}}\right)\mathrm{dS}+\\ +\frac{\beta \nu }{h}\int_{\Gamma_{D}}\left(v-v_{\mathrm{bc}}\right)\cdot w\;\mathrm{dS} \end{array}$$

where h denotes the circumdiameter, that is the diameter of a circumscribed sphere for each cell, and β is a parameter that needs to be adjusted for the particular problem.

The finite element choice is crucial in obtaining stable numerical solutions. Selecting an inf‐sup stable finite element pair is necessary [22], or, alternatively, implementing stabilization techniques when the pair lacks inherent inf‐sup stability. In this study, we focus on two finite elements: the inherently inf‐sup stable Taylor–Hood P2/P1 and the P1/P1 stabilized finite element pairs.

### 2.2 Navier–Stokes Flow

The blood flow within a vessel is governed by the incompressible Navier–Stokes equations under the usual assumptions that the vessel walls are rigid and impermeable, and that volume forces, such as gravitational effects, are neglected. The problem follows

(5a) $$\begin{array}{cc} \rho \frac{\partial v}{\partial t}+\rho \left(\nabla v\right)v=\mathrm{div}T\left(v,p\right) & \;\text{in}\;\Omega_{T} \end{array}$$

(5b) $$\begin{array}{cc} T\left(v,p\right)=-pI+2\mu D & \text{in}\;\Omega_{T} \end{array}$$

(5c) $$\;\begin{array}{cc} \mathrm{div}\;v=0 & \;\text{in}\;\Omega_{T} \end{array}$$

(5d) $$\;\begin{array}{cc} v\left(0,x\right)=v_{0} & \;\text{in}\;\Omega \end{array}$$

(5e) $$\;\begin{array}{cc} v=v_{\mathrm{bc}} & \;\mathrm{on}\;\Gamma_{D}^{T} \end{array}$$

(5f) $$\begin{array}{cc} T\left(v,p\right)n=0 & \;\mathrm{on}\;\Gamma_{N}^{T} \end{array}$$

where ρ is the density and μ the dynamic viscosity of the fluid; t denotes time; T denotes the Cauchy stress tensor, where I is the identity matrix in two or three dimensions, and $D=\frac{1}{2}\left(\nabla v+{\left(\nabla v\right)}^{T}\right)$ is the symmetric part of the velocity gradient. One often assumes that the fluid starts at rest, that is $v_{0}=0$, the inflow velocity is prescribed on the inlet part of the domain; the no‐slip BC is set on walls, and the do‐nothing BC on all outlets. The computational domain Ω in time is denoted $\Omega_{T}\overset{\mathrm{def}\;}{=}\left(0,T\right)\times \Omega$, since the simulation starts at t=0 and ends at t=T. Similarly, we denote the Dirichlet and Neumann boundaries in time by $\Gamma_{D}^{T}$ and $\Gamma_{N}^{T}$, respectively.

The approximate solution of the problem (5) must be sought in a weak sense and discretized in both space and time. Function spaces for velocity and pressure are defined similarly as in the Stokes example: $V^{v_{\mathrm{bc}}}\overset{\mathrm{def}\;}{=}\left\{v\in W^{1,2}\left(\Omega \right)\;|\;v=v_{\mathrm{bc}}\;\mathrm{on}\;\Gamma_{D}^{T}\right\}$ using strong enforcement of Dirichlet BC, while $V\overset{\mathrm{def}\;}{=}\left\{v\in W^{1,2}\left(\Omega \right)\right\}$ for Nitsche enforcement; and $Q\overset{\mathrm{def}\;}{=}L^{2}\left(\Omega \right)$. By employing integration by parts, the corresponding weak formulation with Dirichlet BCs enforced weakly via Nitsche's method yields

(6) $$\begin{array}{c} \begin{array}{l} \text{Find}\;\left(v,p\right)\in V\times Q\;\text{such}\;\text{that}:\\ \;\rho \int_{\Omega }\left(\frac{\partial v}{\partial t}+\left(\nabla v\right)v\right)\cdot w\;\mathrm{dx}+\int_{\Omega }T:\nabla w\;\mathrm{dx}+\int_{\Omega }q\mathrm{div}\;v\;\mathrm{dx}+\\ \;+N_{\Gamma_{D}}\left[\left(v_{\mathrm{bc}};v,p\right),\left(w,q\right)\right]+S\left[\left(v,p\right),\left(w,q\right)\right]=0\;a.e.\;\text{in}\;\left(0,T\right)\;\forall \left(w,q\right)\in V^{0}\times Q \end{array} \end{array}$$

### 2.3 Wall Shear Stress Evaluation

WSS is defined as the tangential component of the traction force per unit area exerted by a flowing fluid on a boundary. In the SI unit system, WSS is measured in pascals (Pa). However, it is most commonly treated as a vectorial quantity, representing both the magnitude and direction of the tangential force. By introducing the notation for tangential part of a vector $a_{t}=a-\left(a\cdot n\right)n$, the definition of WSS, which will be denoted τ hereafter, follows

(7) $$\tau \overset{\mathrm{def}\;}{=}{\left(Tn\right)}_{t}={\left(\left(-pI+2\mu D\right)n\right)}_{t}$$

Usually, the definition of WSS in the literature excludes the pressure term, as this component vanishes when the tangential projection is applied, as shown in Equation (8). Since the Cauchy stress tensor for the 2D Stokes problem is defined using the full velocity gradient, the corresponding wall shear stress formulation is adapted accordingly $\tau \overset{\mathrm{def}\;}{=}{\left(Tn\right)}_{t}={\left(\left(-pI+\mu \nabla v\right)n\right)}_{t}$.

#### 2.3.1 WSS Computation by Projection

Following the definition, WSS is typically numerically evaluated by performing an $L_{2}$ projection to an appropriate function space S

(8) $$\begin{array}{c} \begin{array}{c} \int_{\Gamma_{\text{wall}}}\tau \cdot \phi \;\mathrm{dS}=\int_{\Gamma_{\text{wall}}}\left(Tn-\left(Tn\cdot n\right)n\right)\cdot \\ \;\phi \;\mathrm{dS}=2\mu \int_{\Gamma_{\text{wall}}}\left(\left(Dn\right)-\left(Dn\cdot n\right)n\right)\cdot \phi \;\mathrm{dS}\;\forall \phi \in S \end{array} \end{array}$$

The function space for the above test functions, as well as for WSS, is often set to vectorial P1 space. For example, this approach is implemented in the open‐source VaMPy package [23] and the SimVascular software [24]. Commercial software typically does not specify the computational methods used; however, output data appear to be continuous and linear, as indicated by studies utilizing COMSOL Multiphysics [25] and STAR‐CCM+ [26], both of which are based on FEM. Although the P1 space seems like an intuitively good option, the continuity of the velocity field across elements does not ensure the continuity of velocity gradients or traction forces. Therefore, we hypothesize that utilizing discontinuous Galerkin polynomial function spaces may offer benefits for WSS computation. Depending on the choice of finite element (FE) space for velocities, the natural discontinuous space for WSS should be of one order less than for velocity. For instance, DG‐0 WSS when using P1/P1 stabilized element, while DG‐1 for P2/P1 element. Additionally, a significant benefit of this approach is that the projection mass matrix can be inverted locally, as there is no coupling across elements. This local inversion can substantially accelerate computations on fine meshes, a benefit particularly relevant to applications with moving domains like fluid–structure interaction problems, where the mass matrix must be recomputed with each mesh movement.

Moreover, it is important whether the projection is done only on the boundaries of the domain $S=S\left(\Gamma_{\text{wall}}\right)$ or in the entire domain $S=S\left(\Omega \right)$. In the latter case, one commonly sets WSS to zero at all interior degrees of freedom (DOFs). This adjustment does not follow from the formulation above, as those DOFs are not part of the equation. Without this notion, the corresponding matrix A would be singular. It is clear that the test functions will be different depending on the space S, which can especially influence results in corner elements.

#### 2.3.2 Boundary‐Flux Evaluation of WSS

The boundary‐flux evaluation technique was first introduced by Carey in 1985 for the Poisson problem [13] and was later extended by Shakib in 1991 [15] to tackle compressible Euler and compressible Navier–Stokes equations, and by van Brummelen in 2011 to address boundary‐coupled problems [14]. This technique enables the calculation of fluxes or stresses directly from finite element solutions. It is an essential technique, for example, in fluid–structure interaction problems where the flux evaluation appears implicitly in the problem formulation, and it has a direct connection to methods using Lagrange multipliers to enforce Dirichlet boundary conditions [27, 28]. In our case, the stress on the boundary is reconstructed from a solution to the Navier–Stokes equations as a post‐processing operation. Our approach follows the same idea of variationally consistent calculation of boundary fluxes that has been described in context of fluid‐structure interaction, for example, in [29].

Assume that a weak solution $\left(v,p\right)$ to Equation (3) has been obtained under Dirichlet BCs applied to all boundaries, resulting in $\Gamma_{N}=\emptyset$. The objective is to determine the flux through the walls that upholds the no‐slip boundary condition. This flux is identified with the traction force on the boundary, denoted by $t=T\left(v,p\right)n$. Mathematically, this approach is equivalent to prescribing Neumann BC in place of Dirichlet BC on all boundaries and subsequently evaluating the force on the boundary using the known solution $\left(v,p\right)$; see [13, 14, 15] for further details. Moreover, let us assume that the Dirichlet BC was imposed strongly in Equation (3). The term $N_{\partial \Omega }\left[\left(v_{\mathrm{bc}};v,p\right),\left(w,q\right)\right]$ therefore vanishes and the boundary‐flux evaluation for traction force reads

(9) $$\begin{array}{c} \int_{\partial \Omega }t\cdot \phi \;\mathrm{dS}=\int_{\Omega }T\left(v,p\right):\nabla \phi \;\mathrm{dx}-\int_{\Omega }f\cdot \phi \;\mathrm{dx}\\ +S\left[\left(v,p\right),\left(\phi ,0\right)\right]\;\forall \phi \in V \end{array}$$

It should be noted that the term associated with the test function from pressure space, q, is set to zero due to the absence of a corresponding term on the left‐hand side. Furthermore, the objective is to compute WSS, which is solely the tangential component of t that will be denoted by $\tau_{b}$ below. By subtracting the normal traction from both sides of Equation (9), one obtains the boundary‐flux evaluation for WSS as follows

(10) $$\begin{array}{c} \int_{\partial \Omega }\tau_{b}\cdot \phi \;\mathrm{dS}=\int_{\Omega }T\left(v,p\right):\nabla \phi \;\mathrm{dx}-\int_{\Omega }f\cdot \phi \;\mathrm{dx}+S\left[\left(v,p\right),\left(\phi ,0\right)\right]\\ -\int_{\partial \Omega }\left(T\left(v,p\right)n\cdot n\right)n\cdot \phi \;\mathrm{dS}\;\forall \phi \in V \end{array}$$

Subsequently, we turn our attention to the problem (5), with the aim of evaluating WSS only on walls. We denote the inlet surface by $\Gamma_{\text{in}}$, the outlet parts by $\Gamma_{\text{out}}$ and the walls by $\Gamma_{\text{wall}}$. Consequently, the right‐hand side must include both the time derivative term and the convective term. Moreover, the following Neumann boundary terms corresponding to the remaining boundary segments, $\Gamma_{\text{in}}$ and $\Gamma_{\text{out}}$, must be incorporated into the right‐hand side of the boundary‐flux evaluation, regardless of the original BC type

(11) $$\begin{array}{c} \begin{array}{l} \int_{\Gamma_{\text{wall}}}\tau_{b}\cdot \phi \;\mathrm{dS}=\rho \int_{\Omega }\left(\frac{\partial v}{\partial t}+\left(\nabla v\right)v\right)\cdot \phi \;\mathrm{dx}+\int_{\Omega }T\left(v,p\right):\nabla \phi \;\mathrm{dx}\\ -\int_{\Omega }f\cdot \phi \;\mathrm{dx}+S\left[\left(v,p\right),\left(\phi ,0\right)\right]\;-\int_{\Gamma_{\text{wall}}}\left(T\left(v,p\right)n\cdot n\right)n\cdot \phi \;\mathrm{dS}\\ -\int_{\Gamma_{\text{in}}}T\left(v,p\right)n\cdot \phi \;\mathrm{dS}-\int_{\Gamma_{\text{out}}}T\left(v,p\right)n\cdot \phi \;\mathrm{dS}\;\forall \phi \in V \end{array} \end{array}$$

Note that the natural outlet BC as well as the source term f might be omitted thanks to our original problem formulation, and the time derivative term may be omitted as well in the case of steady‐state flow. However, we keep all terms in the formulation for the sake of completeness.

A generalization of the above boundary‐flux evaluation is necessary when the Dirichlet BC on the wall was imposed by Nitsche's method. In that case, two of the Nitsche terms remain on the right‐hand side, while the third term is the unknown flux. The boundary‐flux evaluation for WSS thus reads

(12) Findτb∈L2Γwallsuch that:∫Γwallτb⋅ϕdS=ρ∫Ω∂v∂t+∇vv⋅ϕdx+∫ΩTv,p:∇ϕdx−∫Ωf⋅ϕdx+Sv,p,ϕ0−∫ΓwallTv,pn⋅nn⋅ϕdS+∫ΓwallTϕ0n⋅v−vbcdS+βμh∫Γwallv−vbc⋅ϕdS−∫ΓinTv,pn⋅ϕdS−∫ΓoutTv,pn⋅ϕdS∀ϕ∈V

## 3 Methods

For the projection method, three different finite element spaces for WSS are considered: the DG‐0, DG‐1, and P1 spaces. For the boundary‐flux evaluation, we consider P1 and second‐order Lagrange (P2) spaces, depending on the mixed FE space.

As mentioned above, two types of finite element pairs for velocity and pressure are investigated: the Taylor–Hood P2/P1 element and stabilized P1/P1 element. The Taylor–Hood P2/P1 element satisfies the inf‐sup condition [22] and therefore does not require additional stabilization; however, the computational cost is demanding. In contrast, the P1/P1 element is computationally cheaper, but it violates the inf‐sup condition and therefore needs to be stabilized; the specific stabilization method depends on the problem at hand, as described below. It should be noted that both the Taylor–Hood P2/P1 and stabilized P1/P1 elements do not enforce pointwise incompressibility, unlike divergence‐free elements such as the Scott–Vogelius. However, the Scott–Vogelius element is less practical due to its higher computational costs and stricter mesh requirements (its stability depends on specific mesh structures, such as barycenter refinement, which can create poorly conditioned elements). The Taylor–Hood and P1/P1 elements remain widely used in the community, Taylor–Hood for its stability and good pressure approximation and P1/P1 for its simplicity and low computational cost, making both suitable for large‐scale 3D simulations.

The accuracy of the aforementioned methods and finite element choices is assessed using two academic examples with known solutions and one real‐world example. The first example involves stationary Stokes flow in a 2D unit square domain, and the second examines 3D Poiseuille flow through a cylindrical pipe. In these cases, the exact solutions are known, allowing for the assessment of convergence rates by measuring the relative errors with respect to the exact solutions. The final example focuses on stationary Navier–Stokes flow in 3D image‐based aneurysm geometries. Since exact solutions are not available for these scenarios, the maximum, minimum, and average WSS values over the aneurysm dome, together with the low shear area (LSA) indicator, are shown for various mesh sizes.

### 3.1 Implementation

All computational codes used in this study are publicly available on Zenodo [30]. Additionally, all computational meshes can be found at Zenodo [31], which ensures the reproducibility of our results. A monolithic scheme is utilized to obtain numerical solutions of Stokes and Navier–Stokes simulations, implemented with the FEniCS library [16] using a nonlinear solver from the PETSc library [32]. The boundary quantities are treated as variables defined over the entire domain, with values set to zero at all interior DOFs.

### 3.2 2D Stokes Flow

We base the 2D Stokes flow on an analytical solution presented by Burman and Hansbo [33]. The exact solution is given by $v=\left(20{\mathrm{xy}}^{3},5x^{4}-5y^{4}\right)$ and $p=60x^{2}y-20y^{3}-5$. A suitable stabilization for the Stokes flow has been described in the aforementioned study [33], which we adopt for both 2D and 3D Stokes flow simulations in this work. The pressure term penalizes jumps in gradients of pressure, while the velocity term imposes penalty for discontinuities in divergence of velocity across elements. It reads

$$S\left[\left(v,p\right),\left(w,q\right)\right]=j\left(p,q\right)+\tilde{j}\left(v,w\right)$$

(13) $$j\left(p,q\right)=\sum_{K}\frac{1}{2}\int_{\partial \Omega }\gamma_{p}h_{K}^{s+1}〚n\cdot \nabla p〛〚n\cdot \nabla q〛\;\mathrm{dS}$$

(14) $$\tilde{j}\left(v,w\right)=\sum_{K}\frac{1}{2}\int_{\partial \Omega }\gamma_{v}h_{K}^{s+1}〚\nabla \cdot v〛〚\nabla \cdot w〛\;\mathrm{dS}$$

where K denotes a simplex in a discretized computational domain, $〚x〛$ denotes the jump of quantity x over an interior facet ∂K, $〚x〛=0$ for all exterior facets. The stabilization weights were set to $\gamma_{p}=\gamma_{v}=10^{-2}$. The coefficient s is defined as

$$s=\left\{\begin{array}{c} 2\;\text{if}\;\nu \geq h\\ 1\;\text{if}\;\nu <h \end{array}\right.$$

Unit square meshes were generated in FEniCS using $N_{i}=2^{3+i},i=0,\ldots ,6,$ elements across each side, ensuring symmetry. When employing P2/P1 elements, we impose the velocity boundary conditions strongly. However, when using P1/P1 with stabilization, it is important to impose the Dirichlet condition weakly to achieve optimal convergence [34].

Carey [13] demonstrated a significant decline in the quadratic convergence of boundary fluxes at corner elements in the context of the Poisson equation. Specifically, the convergence rate was reduced from the expected value of 2 to approximately 1.4. To address this issue, our implementation of the boundary‐flux evaluation method is done by computing the WSS separately on each side of the square domain. This involves subtracting the appropriate Neumann boundary integrals, following a similar approach to that presented in Equation (11) for our 3D problem. Finally, the WSS is obtained by summing the contributions from the individual boundary segments.

### 3.3 3D Poiseuille Flow

We examine the Poiseuille flow through a cylindrical pipe of radius R=1 mm and length L=2 mm, which is aligned with the z‐axis. The driving force of the flow is a parabolic velocity profile with a maximum value of 1 m/s prescribed on the circular inlet. Hence, the mean velocity is $\bar{v}=0.5$ m/s. The no‐slip BC is imposed on the wall and do‐nothing BC together with normal outflow at the outlet

(15a) $$\;\begin{array}{cc} v=\left(0,0,2\bar{v}\left(1-{\left(\frac{x}{r}\right)}^{2}-{\left(\frac{y}{r}\right)}^{2}\right)\right) & \;\mathrm{on}\;\Gamma_{\text{in}} \end{array}$$

(15b) $$\;\begin{array}{cc} v=0 & \;\mathrm{on}\;\Gamma_{\text{wall}} \end{array}$$

(15c) $$\;\begin{array}{cc} T\left(v,p\right)n=0 & \;\mathrm{on}\;\Gamma_{\text{out}} \end{array}$$

(15d) $$\;\begin{array}{cc} v_{t}=0 & \;\mathrm{on}\;\Gamma_{\text{out}} \end{array}$$

The Cauchy stress tensor for an incompressible fluid flow contains the symmetric part of the velocity gradient, $T\left(v,p\right)=-pI+2\nu D$, rather than the complete velocity gradient, as is the case in 2D Stokes flow. To simulate the Poiseuille flow, it is essential to impose a boundary condition that restricts flow at the outlet to normal components only. For a geometry as simple as the one considered here, this is achieved by applying a Dirichlet boundary condition with zero values for the x‐ and y‐components of velocity. The stabilization weights for P1/P1 element were adjusted for this example and set to $\gamma_{p}=1;\gamma_{v}=10^{-3}$.

It can be shown that the analytical solution for WSS reads

(16) $$\tau_{\text{exact}}=\frac{2\mu u_{m}}{R}=8\mathrm{Pa}$$

where the maximum velocity is $u_{m}=1$ m/s and the dynamic viscosity ν=4mPa⋅s

In this study we used two types of tetrahedral meshes. The uniform mesh was defined by a single global edge length. The boundary layer mesh used the same surface discretization but incorporated four refined near‐wall layers. These layers had an initial height of 10% of the surface edge length and a growth ratio of 1.1, transitioning into a uniform core volume mesh consistent with the global edge length. Such meshes for studying 3D Poiseuille flow were generated using COMSOL Multiphysics 6.1 (www.comsol.com). A sensitivity analysis was performed using both uniform and boundary‐layer meshes with P1/P1 stabilized and P2/P1 elements.

For verification, we compare our results with those obtained using COMSOL Multiphysics 6.1, a commercial software. In COMSOL Multiphysics, we use the Creep Flow physics module, which corresponds to Stokes flow. We apply the same boundary conditions as previously described and use a direct solver to obtain the velocity and pressure fields. We then evaluate the WSS by multiplying the viscosity by the shear rate, which is automatically computed by the software.

### 3.4 Image‐Based Simulations

**FIGURE 1:**
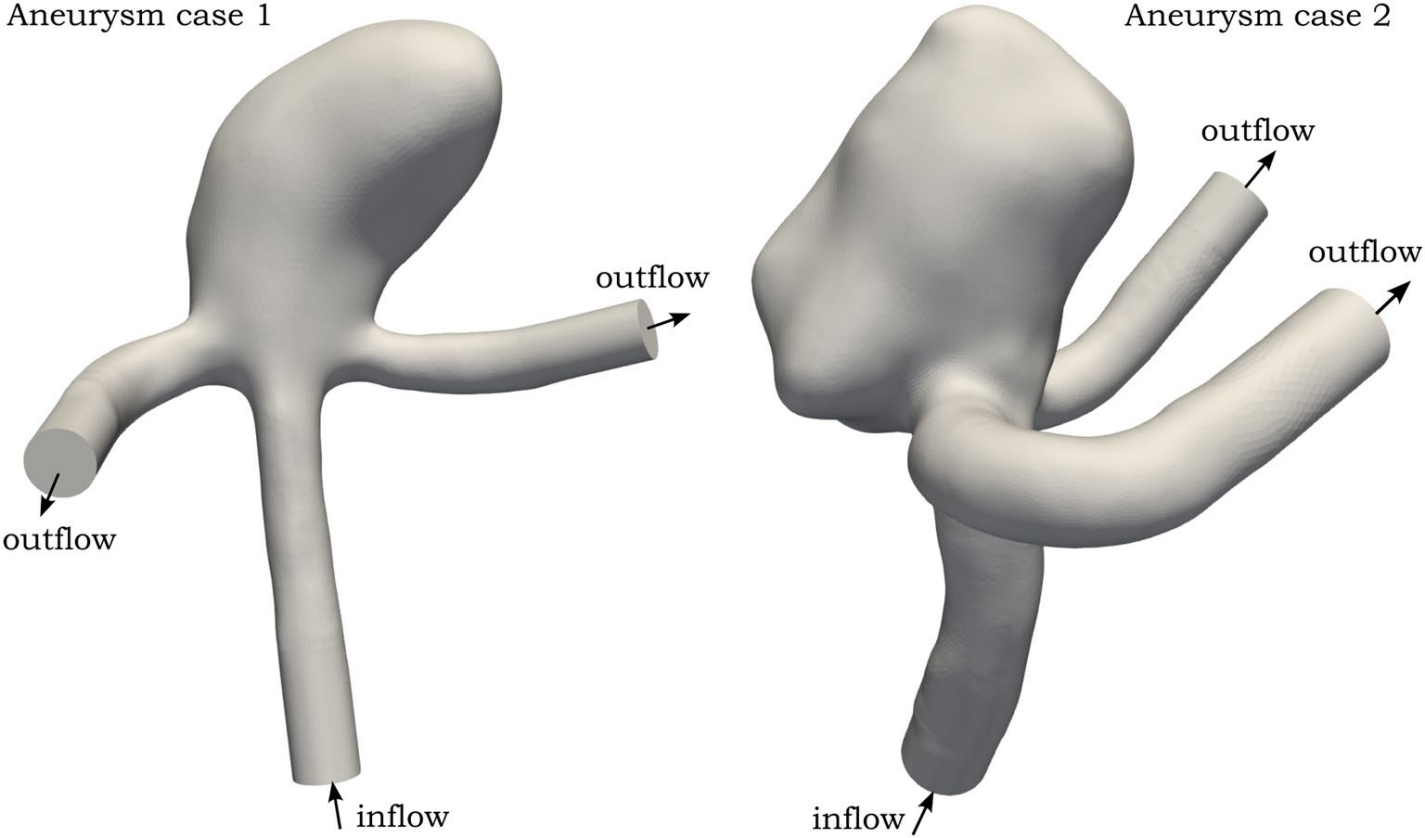
Computational geometries for both aneurysm cases considered in this study, with the inflow branch and two outflow branches indicated by arrows.

Two image‐based patient‐specific aneurysm geometries were adopted from [35]; both aneurysm geometries can be seen in Figure 1. Surface remeshing for these geometries was performed using the PyMeshlab [36] package, achieving the target surface edge length, which ranged from 0.3 mm for the coarsest mesh to 0.1 mm for the finest mesh. Cylindrical flow extensions were added to both inflow and outflow branches. Subsequently, the open‐source GMSH library [37] is employed to generate volume meshes consistent with the previous example, producing one mesh type with boundary layer refinement and another without. The number of boundary layers is again set to four, with the first layer height at 10% of the surface edge length and each subsequent layer increasing by 10%.

Although the numerical solver is inherently designed for time‐dependent simulations, a steady‐state solution can be found using a suitable adaptive time‐stepping. In particular, we use the BDF2 time‐stepping with adaptivity in time steps between $10^{-2}-10^{5}$. Initially, the velocity field is set to zero throughout the domain. Subsequently, the inflow velocity is linearly ramped up over a period of 0.5 s to initiate the desired inflow, which is parabolic velocity profile with a mean value of 0.5 m/s, consistent with [38]. The corresponding Reynolds numbers are 269 for aneurysm case 1 and 263 for aneurysm case 2. Do‐nothing BC is used on all outlets. The total simulation length is set to an arbitrarily large value; we chose $T=10^{6}$ seconds, as done in a previous study [39]. These settings ensure that the solution effectively reaches a steady state since the time derivative term must be of order less than $10^{-5}$ when the largest timestep is used at the end of the simulation.

For P1/P1 element, we use the interior penalty stabilization as introduced in [40]

(17) $$\begin{array}{c} S\left[\left(v,p\right),\left(w,q\right)\right]=\\ \sum_{K\in T_{h}}\left[\alpha_{i}h^{2}\int_{\partial K}{\left|v\cdot n\right|}^{2}〚\nabla v〛〚\nabla w〛\mathrm{dS}+\alpha_{v}h^{2}\int_{\partial K}〚\nabla v〛〚\nabla w〛\mathrm{dS}+\alpha_{p}h^{2}\int_{\partial K}〚\nabla p〛〚\nabla q〛\mathrm{dS}\right] \end{array}$$

where the weights were chosen in accordance with the Poiseuille flow problem above, $\alpha_{v}=10^{-3},\alpha_{p}=1$, and $\alpha_{i}=10^{-3}$.

For each finite element selection, the mean WSS across the aneurysm dome is assessed using both the projection method and the boundary‐flux evaluation technique, as described in Sections 2.3.1 and 2.3.2, respectively. Again, we consider the DG‐0, DG‐1 and P1 spaces for the projection method. Subsequently, for each WSS evaluation method we compute the maximum, minimum and average WSS values over the aneurysm dome as well as the LSA indicator. The LSA is defined as the percentage of the aneurysm wall area exposed to WSS below 10% of the mean WSS in the parent artery, as introduced in [41].

Post‐processing computations are performed using the FEniCS library [16]; interactive mesh clipping and surface plots are conducted in ParaView (www.paraview.org).

## 4 Results

### 4.1 2D Stokes Flow

**FIGURE 2:**
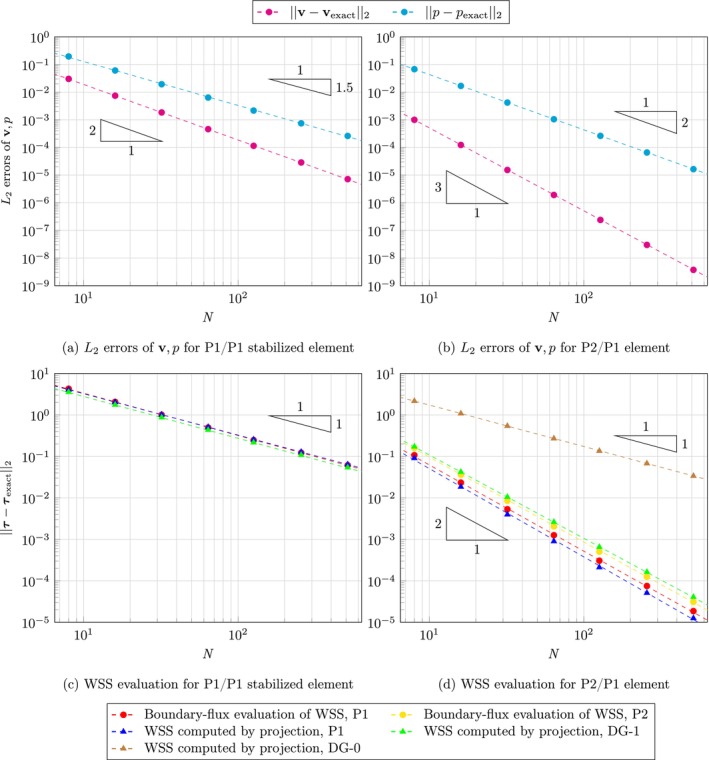
2D Stokes flow: Logarithmic plot of $L_{2}$ error in velocity, pressure, and WSS as a function of number of elements across the side of the square, with errors evaluated against the analytical solution. Red and yellow circles show boundary‐flux evaluation; green, blue and brown triangles show WSS computed by projection in P1, DG‐1 and DG‐0 space, respectively. Dashed lines represent computed convergence rate for corresponding datasets. (a) $L_{2}$ errors of v,p for P1/P1 stabilized element. (b) $L_{2}$ errors of v,p for P2/P1 element. (c) WSS assessment for P1/P1 stabilized element. (d) WSS assessment for P2/P1 element.

Figure 2 shows $L_{2}$ errors of velocity and pressure, together with all considered WSS assessment methods. Errors were calculated with respect to analytical solutions. When using P1/P1 stabilized element, the convergence rate is 2 for velocity and 1.59 for pressure, see Figure 2a. While for P2/P1 element, a convergence rate of 3 is achieved for velocity and 2 for pressure as shown in Figure 2b. WSS assessment for P1/P1 stabilized element yielded linear convergence and the differences between the methods are very small. For P2/P1 element, the convergence is quadratic for both boundary‐flux evaluation and standard evaluation (DG‐1 or P1). However, only linear convergence is achieved for DG‐0 WSS.

### 4.2 3D Poiseuille Flow

**FIGURE 3:**
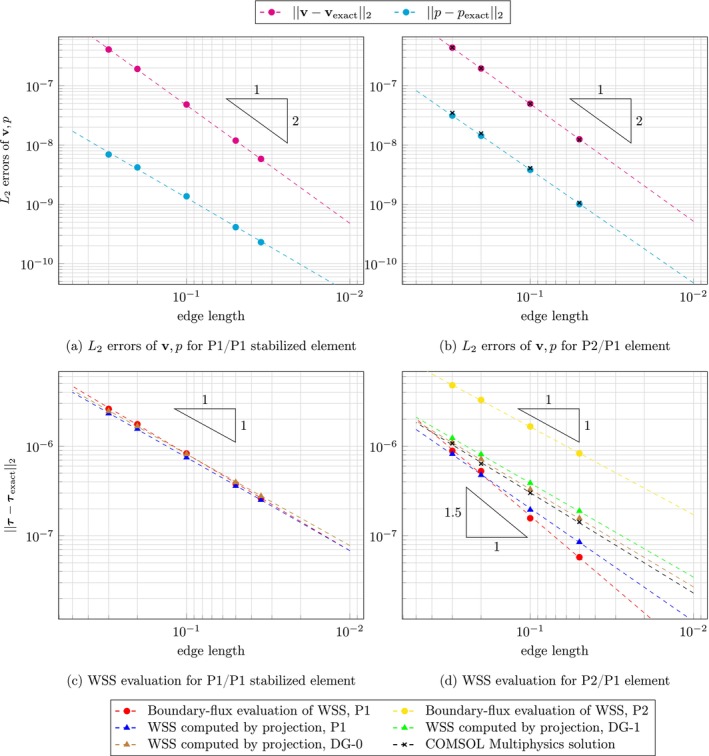
3D Poiseuille flow using uniform meshes: Logarithmic plot of $L_{2}$ error in velocity, pressure, and WSS as a function of edge length, with errors evaluated against the analytical solution. Red and yellow circles show boundary‐flux evaluation; green, blue and brown triangles show WSS computed by projection in P1, DG‐1 and DG‐0 space, respectively. Dashed lines represent computed convergence rate for corresponding datasets. (a) $L_{2}$ errors of v,p for P1/P1 stabilized element. (b) $L_{2}$ errors of v,p for P2/P1 element. (c) WSS assessment for P1/P1 stabilized element. (d) WSS assessment for P2/P1 element.

**FIGURE 4:**
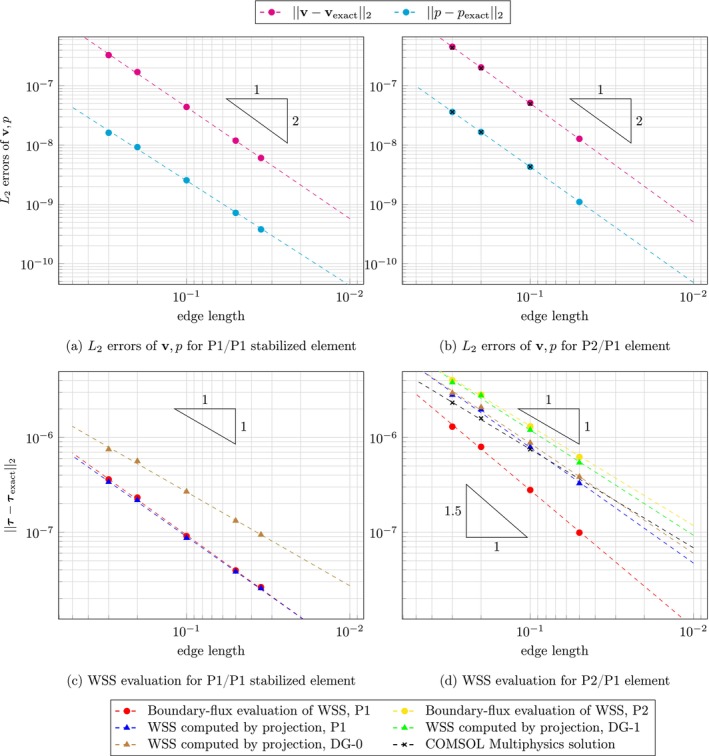
3D Poiseuille flow using meshes with boundary layers: Logarithmic plot of $L_{2}$ error in velocity, pressure, and WSS as a function of edge length, with errors evaluated against the analytical solution. Red and yellow circles show boundary‐flux evaluation; green, blue and brown triangles show WSS computed by projection in P1, DG‐1 and DG‐0 space, respectively. Dashed lines represent computed convergence rate for corresponding datasets. (a) $L_{2}$ errors of v,p for P1/P1 stabilized element. (b) $L_{2}$ errors of v,p for P2/P1 element. (c) WSS assessment for P1/P1 stabilized element. (d) WSS assessment for P2/P1 element.

Figure 3 shows $L_{2}$ errors of velocity, pressure and WSS with respect to analytical solutions using uniform meshes, whereas Figure 4 displays results for meshes with boundary layers. Since the momentum equation is divided by density, as is typical for Stokes problems, it should be noted that both the pressure and WSS values presented in the Poiseuille flow example are scaled by density.

Using uniform meshes, the convergence rate for velocity is 1.99 and 1.61 for pressure in case of P1/P1 stabilized element. Using P2/P1 element and our open‐source FEniCS code, velocity and pressure converge with rates of 1.98 and 1.91, respectively, and COMSOL Multiphysics solutions give very similar rates of 2.00 and 1.95, respectively. Using meshes with boundary layers and P1/P1 stabilized element, velocity and pressure convergence rates were 1.88 and 1.78, respectively. For the P2/P1 element, the FEniCS based code gives velocity and pressure that converge with a rate of 1.99 and 1.95, respectively; COMSOL Multiphysics solutions give rates of 1.98 and 1.94, respectively.

WSS evaluation on uniform meshes for the P1/P1 stabilized element yields a rate of 1.08 for the boundary‐flux evaluation, 1.04 for the projection into P1 space, and 1.02 for both projections to DG‐0 and DG‐1. Using meshes with boundary layers, one obtains better results in absolute errors and improved the rates for P1‐1.24 for the boundary‐flux evaluation and 1.22 for the projection into P1 space—while both discontinuous projections give a rate of 0.99. For the P2/P1 element on uniform meshes, a significant improvement in convergence rate is observed for the boundary‐flux evaluation with P1 space, reaching a rate of 1.56, however, using a P2 space gives worse results with a rate of 0.98. The standard evaluation attains a rate of 1.26 using P1 space, while 1.05 and 1.09 for DG‐1 and DG‐0 spaces. WSS evaluated in COMSOL converges with a rate of 1.12. Finally, using meshes with boundary layers and P2/P1 element, boundary‐flux evaluation gives a rate of 1.45 for P1 space, and 1.05 for P2 space. The standard evaluation yields rates of 1.22, 1.11 and 1.16 using P1, DG‐1 and DG‐0 spaces, respectively. COMSOL solution converges with a rate of 1.04. All $L_{2}$ errors of velocity, pressure and WSS, together with corresponding convergence rates, can be found in Tables S1–S4.

### 4.3 Image‐Based Simulations

**FIGURE 5:**
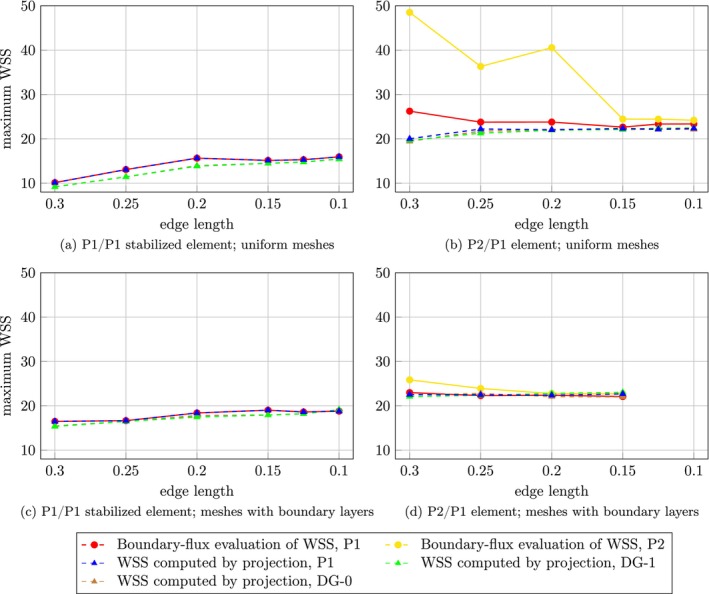
Aneurysm case 1: Mesh sensitivity analysis of the maximum WSS over the aneurysm dome using the P1/P1 stabilized element (left column) and the P2/P1 element (right column) on two mesh types: uniform meshes (top row) and meshes with boundary layers (bottom row).

**FIGURE 6:**
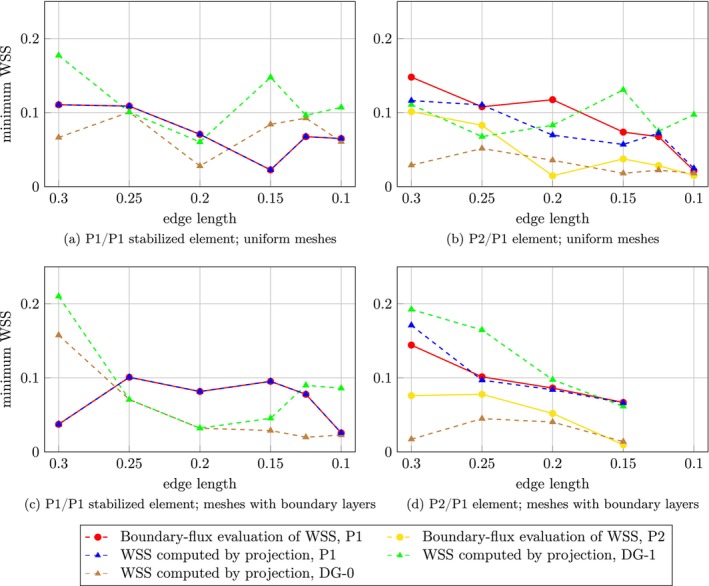
Aneurysm case 1: Mesh sensitivity analysis of the minimum WSS over the aneurysm dome using the P1/P1 stabilized element (left column) and the P2/P1 element (right column) on two mesh types: uniform meshes (top row) and meshes with boundary layers (bottom row).

**FIGURE 7:**
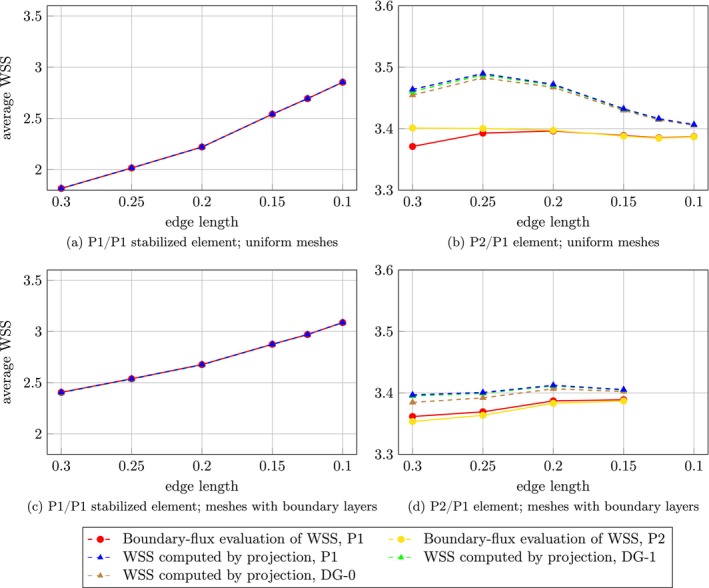
Aneurysm case 1: Mesh sensitivity analysis of the average WSS over the aneurysm dome using the P1/P1 stabilized element (left column) and the P2/P1 element (right column) on two mesh types: uniform meshes (top row) and meshes with boundary layers (bottom row).

**FIGURE 8:**
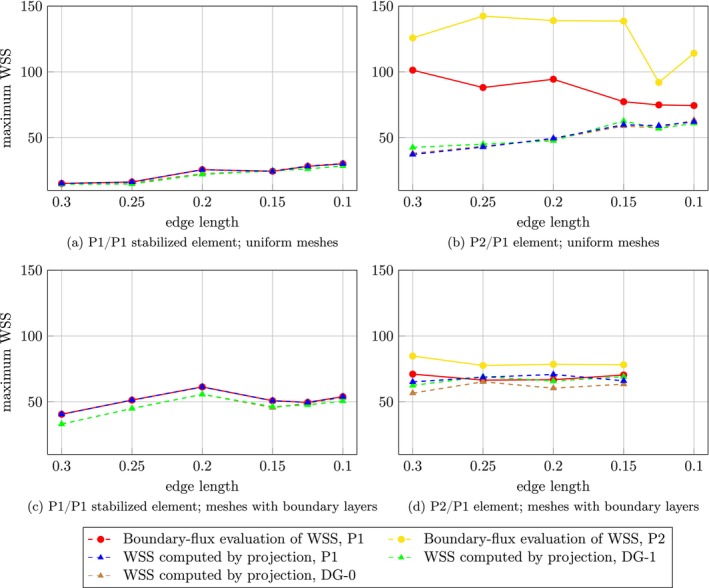
Aneurysm case 2: Mesh sensitivity analysis of the maximum WSS over the aneurysm dome using the P1/P1 stabilized element (left column) and the P2/P1 element (right column) on two mesh types: uniform meshes (top row) and meshes with boundary layers (bottom row).

**FIGURE 9:**
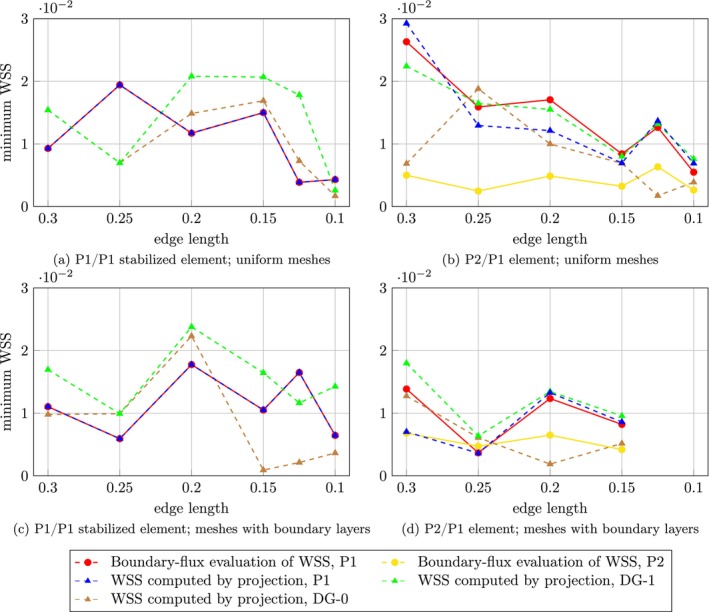
Aneurysm case 2: Mesh sensitivity analysis of the minimum WSS over the aneurysm dome using the P1/P1 stabilized element (left column) and the P2/P1 element (right column) on two mesh types: uniform meshes (top row) and meshes with boundary layers (bottom row).

**FIGURE 10:**
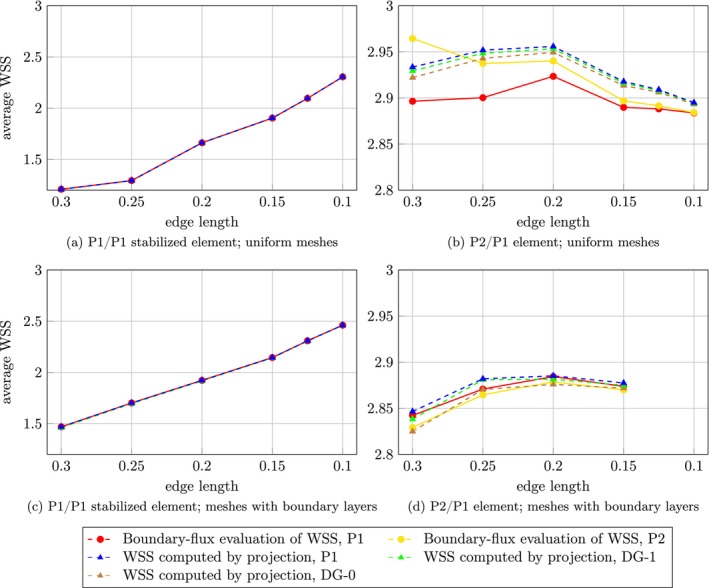
Aneurysm case 2: Mesh sensitivity analysis of the average WSS over the aneurysm dome using the P1/P1 stabilized element (left column) and the P2/P1 element (right column) on two mesh types: uniform meshes (top row) and meshes with boundary layers (bottom row).

Maximum, minimum, and average WSS over the aneurysm dome were assessed for each choice of finite element pair, two mesh types, and for both the projection and the boundary‐flux evaluation method; see Figures 5, 6, 7 for case 1 and Figures 8, 9, 10 for case 2 aneurysm. Additionally, all WSS values are shown in Tables S5–S10. It can be observed that the P2/P1 element is much more robust in assessing the average values of WSS than the P1/P1 stabilized element.

**FIGURE 11:**
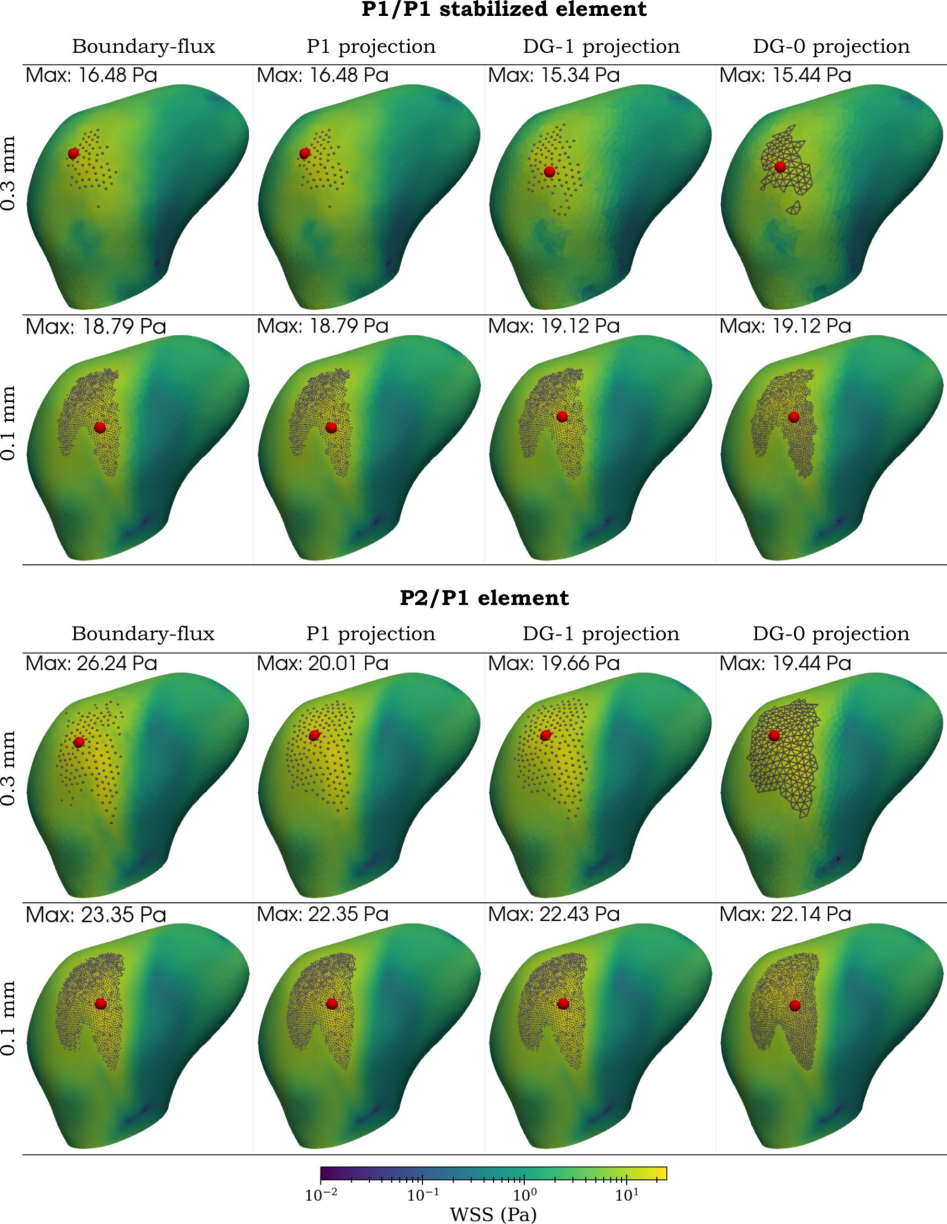
Aneurysm case 1: Comparison of boundary‐flux evaluation and projection method for P2/P1 element. Red points denote the maximum value of WSS over the aneurysm dome, and gray points or triangles represent vertices or facets where WSS≥10 Pa. *Top row*: edge length 0.3 mm; *bottom row*: edge length 0.1 mm.

**FIGURE 12:**
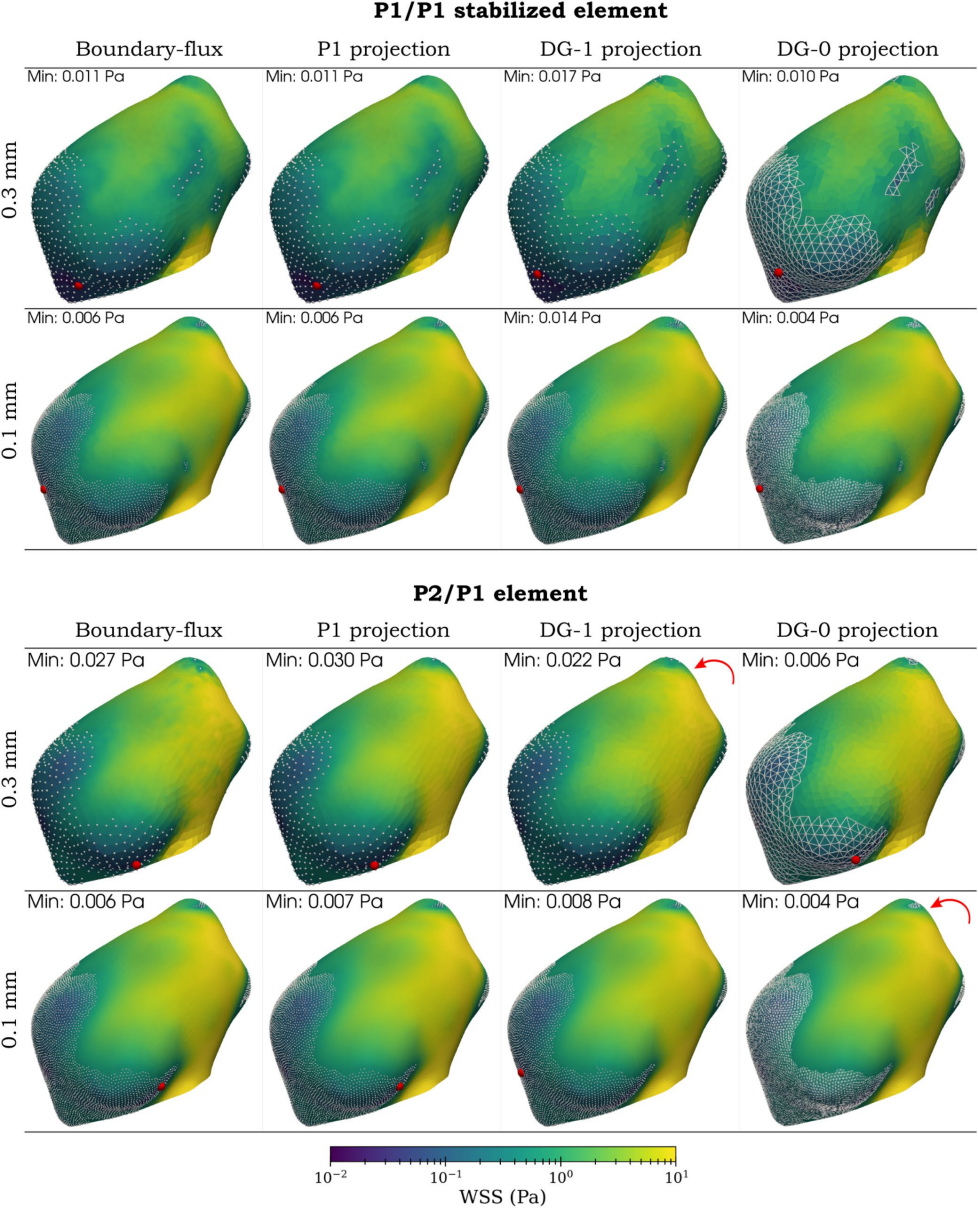
Aneurysm case 2: Comparison of boundary‐flux evaluation and projection method. Red points denote the minimum value of WSS over the aneurysm dome, red arrows indicate the minimum is located outside the current viewpoint; and white points or triangles represent vertices or facets where WSS≤0.5 Pa. *Top row*: edge length 0.3 mm; *bottom row*: edge length 0.1 mm.

Moreover, a visual comparison of all WSS assessment techniques is depicted in Figures 11 and 12. The first of these figures shows the case 1 aneurysm with an annotated maximum WSS over the dome and all boundary vertices (or facets) where WSS is above 10 Pa. The second figure shows the case 2 aneurysm with an annotated minimum value over the dome and all boundary vertices (or facets) where WSS is below 0.5 Pa.

Tables 1 and 2 presents the values of LSA obtained through simulations using the P1/P1 stabilized element and the P2/P1 element, respectively. When using the P1/P1 stabilized element, the LSA indicator exhibits a strong dependence on mesh size, with differences exceeding 100% between the coarsest and finest meshes in case 1 aneurysm. In contrast, the variation between individual evaluation methods is relatively minor (less than 1%). Conversely, for the P2/P1 element, the LSA indicator demonstrates robustness to changes in mesh size. However, in this case, the boundary‐flux evaluation deviates more significantly from the projection methods, with differences up to 5%.

**TABLE 1:** Hemodynamic indicator LSA $\left[\%\right]$ for P1/P1 stabilized element across all WSS evaluation methods, using two different mesh types.

Uniform meshes								
Edge length [mm]	Boundary‐flux P1		P1 projection		DG‐1 projection		DG‐0 projection	
	Case 1	Case 2	Case 1	Case 2	Case 1	Case 2	Case 1	Case 2
0.300	20.44	43.76	0.34	43.71	20.27	43.86	20.27	43.86
0.250	11.84	41.72	11.84	41.50	11.57	41.86	11.57	41.86
0.200	8.12	36.72	8.13	36.71	8.08	36.59	8.08	36.59
0.150	7.08	34.05	7.08	34.04	7.11	33.92	7.11	33.92
0.100	7.04	33.74	7.04	33.74	7.03	33.68	7.03	33.68

Meshes containing boundary layers								
Edge length [mm]	Boundary‐flux evaluation		P1 projection		DG‐1 projection		DG‐0 projection	
	Case 1	Case 2	Case 1	Case 2	Case 1	Case 2	Case 1	Case 2
0.300	16.86	47.83	16.86	47.83	16.76	47.85	16.76	47.85
0.250	11.37	42.46	11.37	42.46	11.46	42.40	11.46	42.40
0.200	9.55	37.96	9.55	37.96	9.46	38.04	9.46	38.03
0.150	8.38	36.05	8.40	36.05	8.31	36.10	8.31	36.10
0.100	8.14	35.00	8.14	34.99	8.12	35.09	8.12	35.09

**TABLE 2:** Hemodynamic indicator LSA $\left[\%\right]$ for P2/P1 element across all WSS evaluation methods, using two different mesh types.

Uniform meshes										
Edge length [mm]	Boundary‐flux P1		Boundary‐flux P2		P1 projection		DG‐1 projection		DG‐0 projection	
	Case 1	Case 2	Case 1	Case 2	Case 1	Case 2	Case 1	Case 2	Case 1	Case 2
0.300	7.82	34.81	8.51	34.89	7.53	33.38	7.49	33.35	7.55	33.21
0.250	7.77	34.79	8.21	34.80	7.75	33.92	7.66	33.84	7.48	33.74
0.200	7.80	34.58	8.06	34.95	7.70	34.33	7.79	34.41	7.64	34.39
0.150	7.80	34.42	7.94	34.69	7.70	34.27	7.67	34.28	7.66	34.26
0.100	7.78	34.49	7.82	34.60	7.68	34.38	7.68	34.41	7.68	34.39

Meshes containing boundary layers										
Edge length [mm]	Boundary‐flux P1		Boundary‐flux P2		P1 projection		DG‐1 projection		DG‐0 projection	
	Case 1	Case 2	Case 1	Case 2	Case 1	Case 2	Case 1	Case 2	Case 1	Case 2
0.300	8.24	34.79	8.37	34.78	8.43	35.11	8.58	35.18	8.21	34.80
0.250	8.20	34.64	8.01	34.46	8.06	34.73	8.17	34.89	7.83	34.62
0.200	7.85	34.65	7.92	34.58	7.86	34.59	7.94	34.70	7.78	34.47
0.150	7.74	34.46	7.72	34.49	7.80	34.53	7.80	34.57	7.70	34.42

## 5 Discussion

### 5.1 2D Stokes Flow

As a proof of concept for the methods presented in this paper, we investigated a 2D Stokes flow on a unit square. The analytical solution is straightforward and it exhibits either constant or linearly increasing behavior along the sides, with a discontinuity located at the upper right corner. Although theoretical predictions indicate quadratic convergence for both velocity and pressure when using the P1/P1 stabilized element [33], tuning the stabilization parameters to achieve this level of convergence proved to be quite challenging. In our experiments, we achieved quadratic convergence for velocity but only a convergence rate of 1.59 for pressure. In contrast, employing the P2/P1 element resulted in convergence rates of 3 for velocity and 2 for pressure, which is in accordance with the theoretical results.

WSS assessment for this problem demonstrated linear convergence for all methods when using the P1/P1 stabilized element, with no significant differences observed in the absolute errors among them. In contrast, when employing the P2/P1 element, linear convergence was achieved only with the DG‐0 projection. For the DG‐1 and P1 projections, as well as boundary‐flux evaluation, quadratic convergence is attained.

It is important to note that convergence rates can be significantly influenced by the nature of the analytical solution. In this example, the analytical WSS on the upper boundary exhibits a linearly increasing behavior, which likely explains why the convergence rate of the DG‐0 projection is one order lower than that of the other methods.

### 5.2 3D Poiseuille Flow

Using meshes with boundary layers improved WSS evaluation in the case of P1/P1 stabilized element; absolute errors were improved for all studied FE spaces and convergence rates were improved when using P1 space (both the projection and boundary‐flux method). Counterintuitively, the same does not hold for the P2/P1 element. We believe this discrepancy arises because the no‐slip condition is enforced on all nodes, including those at the midpoints of element sides on the boundary. Since the cylindrical computational domain is approximated using linear tetrahedral elements, the no‐slip condition introduces an error in the P2 velocity field that subsequently propagates into the WSS calculation via the velocity gradient. Including boundary layers even amplifies this issue. As shown in Figures 3 and 4, the $L^{2}$ velocity error is consistently higher for the P2/P1 discretization than for the stabilized P1/P1 scheme, supporting this explanation.

The discontinuous projection yielded worse results because using discontinuous elements effectively counterweights the aforementioned underlying velocity errors by allowing the WSS solution to jump at each boundary node, even though it ought to be smooth and constant along the wall. We note that further refinement of the surface mesh would help reduce these errors by better approximating the geometry and improving the accuracy of BC enforcement. However, such refinement comes at a substantial computational cost and was therefore not pursued in this study. Similarly, the boundary‐flux evaluation using P2 produced less accurate results than that using P1 due to the same issue. A possible improvement of WSS assessment may include using higher order boundary elements. Although it would be straightforward to do such an improvement of the boundary of a cylinder, there is no simple extension to image‐based geometries, which would limit the applicability of such a method in practice.

### 5.3 Image‐Based Simulations

Using the P1/P1 stabilized element pair, the boundary‐flux evaluation and the P1 projection method yield equivalent results, which is in accordance with the Poiseuille flow example. Discontinuous projections yield differences of maximum values with respect to the continuous projection up to 6.9% (case 1) or 18.5% (case 2) on the coarsest mesh and 1.8% (case 1) or −6.9% (case 2) on the finest mesh. Using the P2/P1 pair, the boundary‐flux evaluation differs from the projection methods, particularly in determining maximum values. The boundary‐flux method seems susceptible to inaccuracies in the pressure field that propagate to the assessment of WSS, unlike the projection method. However, as expected, these discrepancies diminish with mesh refinement.

Furthermore, the results indicate that the minimum WSS value over the aneurysm dome may not be a reliable hemodynamic indicator. Its location shows high sensitivity to both the mesh size and the mixed finite element space employed in the simulation (see Figure 12). This sensitivity affects the consistency and reproducibility of using the minimum WSS value as a hemodynamic indicator in clinical or research applications.

A commonly used hemodynamic indicator derived from WSS is the LSA, which captures the percentage of the aneurysm dome exposed to low wall shear stress (below 10% of the mean value in the parent artery). Our results suggest that this indicator is robust to mesh size when P2/P1 element is used. However, over 100% differences in LSA were observed for P1/P1 stabilized element in aneurysm case 1, which is related to the observation that the average WSS is largely underestimated for coarse meshes, see Figures 7 and 10.

### 5.4 Limitations

As highlighted in the review paper by Steinman et al. [42], numerous factors influence the patient‐specific modeling pipeline, many of which are now well understood and should be standardized. In this context, the most notable limitation of our study is the use of only two anatomically realistic geometries, which precludes any statistical analysis. Additionally, we focused exclusively on steady‐state flows, limiting the flow regime to laminar, although transitional flows could be present in 50% of all aneurysms [43], if care is taken to resolve them [44]. Consequently, we do not claim that the simulations are patient‐specific, although steady‐state simulations have been shown to approximate time‐averaged WSS [5, 45].

### 5.5 Implications and Future Work

From a mathematical perspective, discontinuous spaces for WSS may appear more natural, given the lack of guaranteed continuity in velocity gradients. Although physiological contexts suggest that stresses should ideally be continuous, projecting discontinuous finite element results into continuous spaces, such as P1, can introduce artifacts like the Gibbs phenomenon, as has been previously demonstrated in [46]. Therefore, it warrants attention whether the choice of finite element formulation introduces such artifacts, as this may impact the reliability of the results.

A more general boundary condition than the standard no‐slip condition can be considered for the walls, such as Navier's slip boundary condition. This condition establishes a relationship between the tangential velocity on the boundary and the tangential traction (i.e., WSS). With such a boundary condition, WSS assessment becomes particularly straightforward, as WSS is essentially a rescaled tangential velocity at the boundary, see [21]. Consequently, there is no need to solve an additional system to evaluate WSS. From this perspective, WSS could live in the same finite element space as the velocity field.

## 6 Conclusions

WSS is one of the most commonly studied hemodynamic indicators, although the methods for its numerical evaluation have not yet been studied in detail. This study investigated numerical approaches for evaluating WSS using FEM, using the P1/P1 stabilized and P2/P1 mixed finite elements for velocity and pressure. WSS was evaluated using a boundary‐flux method introduced in this paper and the standard $L_{2}$ projection method applied to three finite element spaces: P1, DG‐1, and DG‐0.

For the P1/P1 stabilized element, the boundary‐flux method and the P1 projection method yielded equivalent results. In contrast, the performance of the P2/P1 element was more complex; while the boundary‐flux method achieved faster convergence in the Poiseuille flow example, it was sensitive to pressure inaccuracies in image‐based geometries, unlike the projection method in which the pressure term vanishes.

Perhaps our most critical finding is the counterintuitive performance of the P2/P1 element on cylindrical meshes that include boundary layer refinement. We observed that adding boundary layers degraded WSS accuracy in all considered evaluation techniques. This phenomenon arises from a geometric approximation error on the curved surface, where the P2 element's mid‐edge velocity nodes lie inside the cylinder. The error of the velocity gradient, and thus WSS, is magnified by the fine near‐wall elements, leading to a less accurate WSS assessment. Ultimately, our work underscores that higher‐order elements do not inherently guarantee superior WSS accuracy. The choice of element, WSS evaluation method, and meshing strategy—particularly near the wall—must be carefully considered, as their interplay can significantly impact the reliability and accuracy of hemodynamic quantities.

## Author Contributions

**Jana Brunátová:** software (equal); visualization (lead); writing – original draft (lead); writing – review and editing (equal). **Jørgen S. Dokken:** conceptualization (equal); software (equal); writing – review and editing (equal). **Kristian Valen‐Sendstad:** conceptualization (equal); writing – review and editing (equal). **Jaroslav Hron:** software (equal); conceptualization (equal); writing – review and editing (supporting).

## Conflicts of Interest

The authors declare no conflicts of interest.

## Supporting information


**Data S1:** Supporting Information.

## Data Availability

The source code for this study is publicly accessible on Zenodo https://doi.org/10.5281/zenodo.14506052 and all computational meshes are available at https://doi.org/10.5281/zenodo.14503385.
