# Supplementary material for: On the Numerical Evaluation of Wall Shear Stress Using the Finite Element Method
Source: Int J Numer Method Biomed Eng. 2025 Sep 12;41(9):e70086. doi: 10.1002/cnm.70086 (PMC12426767; doi:10.1002/cnm.70086)
Supplement: Supplementary file 1 — Data S1: Supporting Information. [file CNM-41-e70086-s001.pdf]

**TABLE S1** 3D Stokes flow on uniform meshes: Velocity and pressure errors in FEniCS and COMSOL.

| | $\|\mathbf{v}-\mathbf{v}_{\text{exact}}\|_2$ | | | $\|p-p_{\text{exact}}\|_2$ | | |
|---|---|---|---|---|---|---|
| **edge length [mm]** | **P1/P1 stab.** | **P2/P1: FEniCS** | **P2/P1: COMSOL** | **P1/P1 stab.** | **P2/P1: FEniCS** | **P2/P1: COMSOL** |
| 0.300 | $4.10 \cdot 10^{-7}$ | $4.39 \cdot 10^{-7}$ | $4.43 \cdot 10^{-7}$ | $6.94 \cdot 10^{-9}$ | $3.14 \cdot 10^{-8}$ | $3.49 \cdot 10^{-8}$ |
| 0.200 | $1.93 \cdot 10^{-7}$ | $1.96 \cdot 10^{-7}$ | $1.98 \cdot 10^{-7}$ | $4.20 \cdot 10^{-9}$ | $1.43 \cdot 10^{-8}$ | $1.58 \cdot 10^{-8}$ |
| 0.100 | $4.84 \cdot 10^{-8}$ | $4.99 \cdot 10^{-8}$ | $4.98 \cdot 10^{-8}$ | $1.37 \cdot 10^{-9}$ | $3.83 \cdot 10^{-9}$ | $4.09 \cdot 10^{-9}$ |
| 0.050 | $1.19 \cdot 10^{-8}$ | $1.25 \cdot 10^{-8}$ | $1.24 \cdot 10^{-8}$ | $4.13 \cdot 10^{-10}$ | $1.01 \cdot 10^{-9}$ | $1.06 \cdot 10^{-9}$ |
| 0.035 | $5.86 \cdot 10^{-9}$ | $6.29 \cdot 10^{-9}$ | – | $2.31 \cdot 10^{-10}$ | $5.17 \cdot 10^{-10}$ | – |
| **convergence rate** | 1.99 | 1.98 | 2.00 | 1.61 | 1.91 | 1.95 |

**TABLE S2** 3D Stokes flow on meshes with boundary layers: Velocity and pressure errors in FEniCS and COMSOL.

| | $\|\mathbf{v}-\mathbf{v}_{\text{exact}}\|_2$ | | | $\|p-p_{\text{exact}}\|_2$ | | |
|---|---|---|---|---|---|---|
| **edge length [mm]** | **P1/P1 stab.** | **P2/P1: FEniCS** | **P2/P1: COMSOL** | **P1/P1 stab.** | **P2/P1: FEniCS** | **P2/P1: COMSOL** |
| 0.300 | $3.30 \cdot 10^{-7}$ | $4.56 \cdot 10^{-7}$ | $4.41 \cdot 10^{-7}$ | $1.62 \cdot 10^{-8}$ | $3.63 \cdot 10^{-8}$ | $3.61 \cdot 10^{-8}$ |
| 0.200 | $1.71 \cdot 10^{-7}$ | $2.04 \cdot 10^{-7}$ | $1.98 \cdot 10^{-7}$ | $9.25 \cdot 10^{-9}$ | $1.66 \cdot 10^{-8}$ | $1.66 \cdot 10^{-8}$ |
| 0.100 | $4.40 \cdot 10^{-8}$ | $5.15 \cdot 10^{-8}$ | $4.98 \cdot 10^{-8}$ | $2.55 \cdot 10^{-9}$ | $4.30 \cdot 10^{-9}$ | $4.30 \cdot 10^{-9}$ |
| 0.050 | $1.19 \cdot 10^{-8}$ | $1.28 \cdot 10^{-8}$ | – | $7.20 \cdot 10^{-10}$ | $1.10 \cdot 10^{-9}$ | – |
| **convergence rate** | 1.88 | 1.99 | 1.98 | 1.78 | 1.95 | 1.94 |

**TABLE S3** 3D Stokes flow: WSS errors evaluated in FEniCS and in COMSOL for P2/P1 element using two types of meshes.

| | **uniform meshes** | | | | | |
|---|---|---|---|---|---|---|
| **edge length [mm]** | **boundary-flux P1** | **boundary-flux P2** | **P1 projection** | **DG-1 projection** | **DG-0 projection** | **COMSOL** |
| 0.300 | $8.88 \cdot 10^{-7}$ | $4.79 \cdot 10^{-6}$ | $8.18 \cdot 10^{-7}$ | $1.23 \cdot 10^{-6}$ | $1.09 \cdot 10^{-6}$ | $1.07 \cdot 10^{-6}$ |
| 0.200 | $5.27 \cdot 10^{-7}$ | $3.28 \cdot 10^{-6}$ | $4.76 \cdot 10^{-7}$ | $8.11 \cdot 10^{-7}$ | $7.18 \cdot 10^{-7}$ | $6.38 \cdot 10^{-7}$ |
| 0.100 | $1.57 \cdot 10^{-7}$ | $1.66 \cdot 10^{-6}$ | $1.95 \cdot 10^{-7}$ | $3.88 \cdot 10^{-7}$ | $3.30 \cdot 10^{-7}$ | $3.01 \cdot 10^{-7}$ |
| 0.050 | $5.77 \cdot 10^{-8}$ | $8.30 \cdot 10^{-7}$ | $8.49 \cdot 10^{-8}$ | $1.89 \cdot 10^{-7}$ | $1.57 \cdot 10^{-7}$ | – |
| **convergence rate** | 1.56 | 0.98 | 1.26 | 1.05 | 1.09 | 1.12 |
| | **meshes containing boundary layers** | | | | | |
| **edge length [mm]** | **boundary-flux P1** | **boundary-flux P2** | **P1 projection** | **DG-1 projection** | **DG-0 projection** | **COMSOL** |
| 0.300 | $1.30 \cdot 10^{-6}$ | $4.02 \cdot 10^{-6}$ | $2.81 \cdot 10^{-6}$ | $3.81 \cdot 10^{-6}$ | $2.95 \cdot 10^{-6}$ | $2.33 \cdot 10^{-6}$ |
| 0.200 | $7.96 \cdot 10^{-7}$ | $2.83 \cdot 10^{-6}$ | $1.97 \cdot 10^{-6}$ | $2.78 \cdot 10^{-6}$ | $2.08 \cdot 10^{-6}$ | $1.59 \cdot 10^{-6}$ |
| 0.100 | $2.79 \cdot 10^{-7}$ | $1.31 \cdot 10^{-6}$ | $7.91 \cdot 10^{-7}$ | $1.20 \cdot 10^{-6}$ | $8.77 \cdot 10^{-7}$ | $7.50 \cdot 10^{-7}$ |
| 0.050 | $9.89 \cdot 10^{-8}$ | $6.22 \cdot 10^{-7}$ | $3.28 \cdot 10^{-7}$ | $5.42 \cdot 10^{-7}$ | $3.84 \cdot 10^{-7}$ | – |
| **convergence rate** | 1.45 | 1.05 | 1.22 | 1.11 | 1.16 | 1.04 |

**TABLE S4** 3D Stokes flow: WSS errors evaluated in FEniCS for P1/P1 stabilized element using two types of meshes.

| | uniform meshes | | | |
|---|---|---|---|---|
| **edge length [mm]** | **boundary-flux P1** | **P1 projection** | **DG-1 projection** | **DG-0 projection** |
| 0.300 | $2.61 \cdot 10^{-6}$ | $2.32 \cdot 10^{-6}$ | $2.46 \cdot 10^{-6}$ | $2.46 \cdot 10^{-6}$ |
| 0.200 | $1.76 \cdot 10^{-6}$ | $1.56 \cdot 10^{-6}$ | $1.67 \cdot 10^{-6}$ | $1.67 \cdot 10^{-6}$ |
| 0.100 | $8.29 \cdot 10^{-7}$ | $7.48 \cdot 10^{-7}$ | $8.14 \cdot 10^{-7}$ | $8.14 \cdot 10^{-7}$ |
| 0.050 | $3.79 \cdot 10^{-7}$ | $3.60 \cdot 10^{-7}$ | $3.96 \cdot 10^{-7}$ | $3.96 \cdot 10^{-7}$ |
| 0.035 | $2.60 \cdot 10^{-7}$ | $2.51 \cdot 10^{-7}$ | $2.77 \cdot 10^{-7}$ | $2.77 \cdot 10^{-7}$ |
| **convergence rate** | 1.08 | 1.04 | 1.02 | 1.02 |
| | **meshes containing boundary layers** | | | |
| **edge length [mm]** | **boundary-flux P1** | **P1 projection** | **DG-1 projection** | **DG-0 projection** |
| 0.300 | $3.61 \cdot 10^{-7}$ | $3.39 \cdot 10^{-7}$ | $7.50 \cdot 10^{-7}$ | $7.50 \cdot 10^{-7}$ |
| 0.200 | $2.32 \cdot 10^{-7}$ | $2.18 \cdot 10^{-7}$ | $5.62 \cdot 10^{-7}$ | $5.62 \cdot 10^{-7}$ |
| 0.100 | $9.11 \cdot 10^{-8}$ | $8.68 \cdot 10^{-8}$ | $2.68 \cdot 10^{-7}$ | $2.68 \cdot 10^{-7}$ |
| 0.050 | $3.95 \cdot 10^{-8}$ | $3.83 \cdot 10^{-8}$ | $1.32 \cdot 10^{-7}$ | $1.32 \cdot 10^{-7}$ |
| 0.035 | $2.63 \cdot 10^{-8}$ | $2.56 \cdot 10^{-8}$ | $9.39 \cdot 10^{-8}$ | $9.39 \cdot 10^{-8}$ |
| **convergence rate** | 1.24 | 1.22 | 0.99 | 0.99 |

**TABLE S5** Maximum values of WSS for P1/P1 stabilized element across various WSS evaluation methods, using two different mesh types.

| | uniform meshes | | | | | | | |
|---|---|---|---|---|---|---|---|---|
| **Edge length** [mm] | **Boundary-flux P1** [Pa] case 1 | case 2 | **P1 Projection** [Pa] case 1 | case 2 | **DG-1 Projection** [Pa] case 1 | case 2 | **DG-0 Projection** [Pa] case 1 | case 2 |
| 0.300 | 16.48 | 40.55 | 16.48 | 40.55 | 15.34 | 33.07 | 15.44 | 33.07 |
| 0.250 | 16.65 | 51.30 | 16.65 | 51.30 | 16.49 | 44.92 | 16.52 | 44.92 |
| 0.200 | 18.38 | 61.32 | 18.38 | 61.32 | 17.47 | 55.67 | 17.79 | 55.67 |
| 0.150 | 19.01 | 50.82 | 19.01 | 50.82 | 17.92 | 46.30 | 17.92 | 45.64 |
| 0.100 | 18.79 | 53.92 | 18.79 | 53.92 | 19.12 | 50.57 | 19.12 | 53.17 |
| | **meshes containing boundary layers** | | | | | | | |
| **Edge length** [mm] | **Boundary-flux P1** [Pa] case 1 | case 2 | **P1 Projection** [Pa] case 1 | case 2 | **DG-1 Projection** [Pa] case 1 | case 2 | **DG-0 Projection** [Pa] case 1 | case 2 |
| 0.300 | 16.48 | 40.55 | 16.48 | 40.55 | 15.34 | 33.07 | 15.44 | 33.07 |
| 0.250 | 16.65 | 51.30 | 16.65 | 51.30 | 16.49 | 44.92 | 16.52 | 44.92 |
| 0.200 | 18.38 | 61.32 | 18.38 | 61.32 | 17.47 | 55.67 | 17.79 | 55.67 |
| 0.150 | 19.01 | 50.82 | 19.01 | 50.82 | 17.92 | 46.30 | 17.92 | 45.64 |
| 0.100 | 18.79 | 53.92 | 18.79 | 53.92 | 19.12 | 50.57 | 19.12 | 53.17 |

**TABLE S6** Maximum values of WSS for P2/P1 element across various WSS evaluation methods, using two different mesh types.

| | uniform meshes | | | | | | | | | |
|---|---|---|---|---|---|---|---|---|---|---|
| **Edge length** [mm] | **Boundary-flux P1** [Pa] case 1 | case 2 | **Boundary-flux P2** [Pa] case 1 | case 2 | **P1 Projection** [Pa] case 1 | case 2 | **DG-1 Projection** [Pa] case 1 | case 2 | **DG-0 Projection** [Pa] case 1 | case 2 |
| 0.300 | 26.24 | 101.33 | 48.51 | 125.82 | 20.01 | 37.27 | 19.66 | 42.61 | 19.44 | 38.12 |
| 0.250 | 23.77 | 88.11 | 36.32 | 142.41 | 22.22 | 42.94 | 21.28 | 45.11 | 21.74 | 43.48 |
| 0.200 | 23.78 | 94.41 | 40.55 | 138.97 | 22.04 | 49.55 | 21.95 | 47.70 | 22.14 | 48.95 |
| 0.150 | 22.64 | 77.34 | 24.45 | 138.58 | 22.33 | 59.86 | 22.11 | 62.65 | 22.09 | 58.92 |
| 0.100 | 23.35 | 74.42 | 24.19 | 114.18 | 22.35 | 62.13 | 22.43 | 60.98 | 22.14 | 63.38 |
| | **meshes containing boundary layers** | | | | | | | | | |
| **Edge length** [mm] | **Boundary-flux P1** [Pa] case 1 | case 2 | **Boundary-flux P2** [Pa] case 1 | case 2 | **P1 Projection** [Pa] case 1 | case 2 | **DG-1 Projection** [Pa] case 1 | case 2 | **DG-0 Projection** [Pa] case 1 | case 2 |
| 0.300 | 22.98 | 71.03 | 25.85 | 84.78 | 22.54 | 65.13 | 22.09 | 62.46 | 22.46 | 56.63 |
| 0.250 | 22.30 | 66.47 | 23.91 | 77.59 | 22.46 | 68.66 | 22.37 | 69.08 | 22.77 | 65.21 |
| 0.200 | 22.38 | 66.78 | 22.75 | 78.51 | 22.38 | 70.79 | 22.77 | 65.76 | 22.09 | 60.34 |
| 0.150 | 22.09 | 70.40 | 22.70 | 78.15 | 22.68 | 65.96 | 23.03 | 69.07 | 22.01 | 63.44 |

**TABLE S7** Minimum values of WSS for P1/P1 stabilized element across various WSS evaluation methods, using two different mesh types.

| Edge length [mm] | Boundary-flux P1 [Pa] | | P1 Projection [Pa] | | DG-1 Projection [Pa] | | DG-0 Projection [Pa] | |
|---|---|---|---|---|---|---|---|---|
| **uniform meshes** | | | | | | | | |
| | case 1 | case 2 | case 1 | case 2 | case 1 | case 2 | case 1 | case 2 |
| 0.300 | 0.111 | 0.009 | 0.111 | 0.009 | 0.177 | 0.015 | 0.066 | 0.015 |
| 0.250 | 0.109 | 0.019 | 0.109 | 0.019 | 0.101 | 0.007 | 0.101 | 0.007 |
| 0.200 | 0.071 | 0.012 | 0.071 | 0.011 | 0.060 | 0.021 | 0.028 | 0.015 |
| 0.150 | 0.023 | 0.015 | 0.023 | 0.015 | 0.148 | 0.021 | 0.084 | 0.017 |
| 0.100 | 0.065 | 0.004 | 0.065 | 0.004 | 0.107 | 0.003 | 0.061 | 0.002 |
| **meshes containing boundary layers** | | | | | | | | |
| | case 1 | case 2 | case 1 | case 2 | case 1 | case 2 | case 1 | case 2 |
| 0.300 | 0.037 | 0.011 | 0.037 | 0.011 | 0.210 | 0.017 | 0.158 | 0.010 |
| 0.250 | 0.101 | 0.006 | 0.101 | 0.006 | 0.071 | 0.010 | 0.071 | 0.010 |
| 0.200 | 0.082 | 0.018 | 0.082 | 0.018 | 0.032 | 0.024 | 0.032 | 0.022 |
| 0.150 | 0.095 | 0.011 | 0.095 | 0.011 | 0.045 | 0.016 | 0.029 | 0.001 |
| 0.100 | 0.026 | 0.006 | 0.026 | 0.006 | 0.086 | 0.014 | 0.023 | 0.004 |

**TABLE S8** Minimum values of WSS for P2/P1 element across various WSS evaluation methods, using two different mesh types.

| Edge length [mm] | Boundary-flux P1 [Pa] | | Boundary-flux P2 [Pa] | | P1 Projection [Pa] | | DG-1 Projection [Pa] | | DG-0 Projection [Pa] | |
|---|---|---|---|---|---|---|---|---|---|---|
| **uniform meshes** | | | | | | | | | | |
| | case 1 | case 2 | case 1 | case 2 | case 1 | case 2 | case 1 | case 2 | case 1 | case 2 |
| 0.300 | 0.148 | 0.027 | 0.101 | 0.005 | 0.116 | 0.029 | 0.111 | 0.022 | 0.029 | 0.007 |
| 0.250 | 0.108 | 0.016 | 0.083 | 0.002 | 0.110 | 0.013 | 0.068 | 0.016 | 0.052 | 0.019 |
| 0.200 | 0.117 | 0.017 | 0.015 | 0.005 | 0.069 | 0.012 | 0.081 | 0.016 | 0.033 | 0.010 |
| 0.150 | 0.074 | 0.008 | 0.037 | 0.003 | 0.057 | 0.007 | 0.131 | 0.008 | 0.018 | 0.007 |
| 0.100 | 0.022 | 0.006 | 0.015 | 0.003 | 0.025 | 0.007 | 0.097 | 0.008 | 0.018 | 0.004 |
| **meshes containing boundary layers** | | | | | | | | | | |
| | case 1 | case 2 | case 1 | case 2 | case 1 | case 2 | case 1 | case 2 | case 1 | case 2 |
| 0.300 | 0.144 | 0.014 | 0.076 | 0.007 | 0.17 | 0.007 | 0.192 | 0.018 | 0.017 | 0.013 |
| 0.250 | 0.101 | 0.004 | 0.078 | 0.005 | 0.097 | 0.004 | 0.165 | 0.006 | 0.045 | 0.006 |
| 0.200 | 0.086 | 0.012 | 0.052 | 0.007 | 0.084 | 0.013 | 0.097 | 0.013 | 0.040 | 0.002 |
| 0.150 | 0.067 | 0.008 | 0.010 | 0.004 | 0.066 | 0.009 | 0.062 | 0.010 | 0.014 | 0.005 |

**TABLE S9** Average values of WSS for P1/P1 stabilized element across various WSS evaluation methods, using two different mesh types.

| Edge length [mm] | Boundary-flux P1 [Pa] | | P1 Projection [Pa] | | DG-1 Projection [Pa] | | DG-0 Projection [Pa] | |
|---|---|---|---|---|---|---|---|---|
| **uniform meshes** | | | | | | | | |
| | case 1 | case 2 | case 1 | case 2 | case 1 | case 2 | case 1 | case 2 |
| 0.300 | 1.82 | 1.21 | 1.82 | 1.21 | 1.82 | 1.21 | 1.82 | 1.21 |
| 0.250 | 2.02 | 1.29 | 2.02 | 1.29 | 2.02 | 1.29 | 2.02 | 1.29 |
| 0.200 | 2.23 | 1.66 | 2.23 | 1.66 | 2.22 | 1.66 | 2.22 | 1.66 |
| 0.150 | 2.54 | 1.90 | 2.54 | 1.90 | 2.54 | 1.90 | 2.54 | 1.90 |
| 0.100 | 2.85 | 2.31 | 2.85 | 2.31 | 2.85 | 2.31 | 2.85 | 2.31 |
| **meshes containing boundary layers** | | | | | | | | |
| | case 1 | case 2 | case 1 | case 2 | case 1 | case 2 | case 1 | case 2 |
| 0.300 | 2.41 | 1.47 | 2.41 | 1.47 | 2.40 | 1.46 | 2.40 | 1.46 |
| 0.250 | 2.54 | 1.70 | 2.54 | 1.70 | 2.54 | 1.70 | 2.54 | 1.70 |
| 0.200 | 2.68 | 1.92 | 2.68 | 1.92 | 2.67 | 1.92 | 2.67 | 1.92 |
| 0.150 | 2.88 | 2.15 | 2.88 | 2.15 | 2.87 | 2.14 | 2.87 | 2.14 |
| 0.100 | 3.09 | 2.46 | 3.09 | 2.46 | 3.09 | 2.46 | 3.09 | 2.46 |

**TABLE S10** Average values of WSS for P2/P1 element across various WSS evaluation methods, using two different mesh types.

| **uniform meshes** | | | | | | | | | | |
|---|---|---|---|---|---|---|---|---|---|---|
| **Edge length** | **Boundary-flux P1** [Pa] | | **Boundary-flux P2** [Pa] | | **P1 Projection** [Pa] | | **DG-1 Projection** [Pa] | | **DG-0 Projection** [Pa] | |
| [mm] | case 1 | case 2 | case 1 | case 2 | case 1 | case 2 | case 1 | case 2 | case 1 | case 2 |
| 0.300 | 3.37 | 2.90 | 3.40 | 2.96 | 3.46 | 2.93 | 3.46 | 2.93 | 3.45 | 2.92 |
| 0.250 | 3.39 | 2.90 | 3.40 | 2.94 | 3.49 | 2.95 | 3.49 | 2.95 | 3.48 | 2.94 |
| 0.200 | 3.40 | 2.92 | 3.40 | 2.94 | 3.47 | 2.96 | 3.47 | 2.95 | 3.47 | 2.95 |
| 0.150 | 3.39 | 2.89 | 3.39 | 2.90 | 3.43 | 2.92 | 3.43 | 2.92 | 3.43 | 2.91 |
| 0.100 | 3.39 | 2.88 | 3.38 | 2.88 | 3.41 | 2.89 | 3.41 | 2.89 | 3.41 | 2.89 |

| **meshes containing boundary layers** | | | | | | | | | | |
|---|---|---|---|---|---|---|---|---|---|---|
| **Edge length** | **Boundary-flux P1** [Pa] | | **Boundary-flux P2** [Pa] | | **P1 Projection** [Pa] | | **DG-1 Projection** [Pa] | | **DG-0 Projection** [Pa] | |
| [mm] | case 1 | case 2 | case 1 | case 2 | case 1 | case 2 | case 1 | case 2 | case 1 | case 2 |
| 0.300 | 3.36 | 2.84 | 3.35 | 2.83 | 3.40 | 2.85 | 3.40 | 2.84 | 3.38 | 2.82 |
| 0.250 | 3.37 | 2.87 | 3.36 | 2.86 | 3.40 | 2.88 | 3.40 | 2.88 | 3.39 | 2.87 |
| 0.200 | 3.39 | 2.88 | 3.38 | 2.88 | 3.41 | 2.89 | 3.41 | 2.88 | 3.41 | 2.88 |
| 0.150 | 3.39 | 2.87 | 3.39 | 2.87 | 3.41 | 2.88 | 3.40 | 2.88 | 3.40 | 2.87 |
